# Diversity of *Methylobacterium* species associated with New Zealand native plants

**DOI:** 10.1093/femsle/fnad124

**Published:** 2023-11-20

**Authors:** Rowshan Jahan, Ian R McDonald

**Affiliations:** Te Aka Mātuatua—School of Science, Te Whare Wānanga o Waikato—University of Waikato, Private Bag 3105, Hamilton 3240, Aotearoa, New Zealand; Te Aka Mātuatua—School of Science, Te Whare Wānanga o Waikato—University of Waikato, Private Bag 3105, Hamilton 3240, Aotearoa, New Zealand

**Keywords:** *Methylobacterium*, phyllosphere, New Zealand, methanol

## Abstract

*Methylobacterium* species are abundant colonizers of the phyllosphere due to the availability of methanol, a waste product of pectin metabolism during plant cell division. The phyllosphere is an extreme environment, with a landscape that is heterogeneous and continuously changing as the plant grows and is exposed to high levels of ultraviolet irradiation. Geographically, New Zealand (NZ) has been isolated for over a million years, has a biologically diverse flora, and is considered a biodiversity hotspot, with most native plants being endemic. We therefore hypothesize that the phyllosphere of NZ native plants harbor diverse groups of *Methylobacterium* species. Leaf imprinting using methanol-supplemented agar medium was used to isolate bacteria, and diversity was determined using ARDRA and 16S rRNA gene sequencing. *Methylobacterium* species were successfully isolated from the phyllosphere of 18 of the 20 native NZ plant species in this study, and six different species were identified: *M. marchantiae, M. mesophilicum, M. adhaesivum, M. komagatae, M. extorquens*, and *M. phyllosphaerae*. Other α, β, and γ-Proteobacteria, Actinomycetes, Bacteroidetes, and Firmicutes were also isolated, highlighting the presence of other potentially novel methanol utilizers within this ecosystem. This study identified that *Methylobacterium* are abundant members of the NZ phyllosphere, with species diversity and composition dependent on plant species.

## Teaser

*Methylobacterium* are abundant members of the New Zealand phyllosphere, with species diversity and composition dependent on plant species.

## Introduction

The phyllosphere (above ground parts of plants comprising mainly stems and leaves) is the Earth’s biggest biological surface, estimated to be two times bigger than the land surface (Woodward and Lomas 2004). The physicochemical environment of the phyllosphere is highly diverse due to fluctuating nutrient and water availability, temperature, wind pressure, exposure to pollutants, UV radiation, and the variable biology of the waxy protective layer (plant cuticle). This diverse environment provides a habitat for many bacteria (Vorholt 2012), with the planetary phyllosphere bacterial population estimated to be as large as 10^26^ cells (Lindow and Brandl 2003). Global processes of carbon, oxygen, and nitrogen cycling are greatly influenced by phyllosphere bacteria, as is plant health, with growth and productivity being enhanced (Trotsenko et al. 2001, Lindow and Brandl 2003). The number and composition of phyllosphere bacteria are greatly influenced by plant species, sampling site, growing season, plant growth stage, location on the plant, leaf properties, and surrounding plant species (Kinkel et al. 2000, Redford et al. 2010, Finkel et al. 2011).

The genus *Methylobacterium* is a dominant group of *Bacteria* in the phyllosphere (Delmotte et al. 2009) and has been estimated at 10^4^–10^7^ cells per gram of fresh plant material (Holland et al. 2002). The *Methylobacterium* association with plants can be epiphytic or endophytic (Corpe and Rheem 1989, Delmotte et al. 2009, Knief et al. 2010); or symbiotic (Sy et al. 2001); however, there are no reports of *Methylobacterium* as the cause of plant disease. The association of *Methylobacterium* spp. with plants typically relies on methanol, a volatile organic compound (VOC), which is released by plants during growth through stomatal pores in the epidermis (Galbally and Kirstine 2002). However, other plant-derived carbon compounds may also support their colonization (Sy et al. 2005, Delmotte et al. 2009). Methanol is produced inside leaves as a byproduct of pectin metabolism during cell wall synthesis. During cell elongation and division pectin methylesterases catalyze the C-6 demethylation of homogalacturonan within plant cell walls, as a result, methanol is released (Korner et al. 2009). The global methanol emission from plants is estimated to be 100–128 Tg per year (Galbally and Kirstine 2002). Colonization of plants by methylotrophic bacteria, especially *Methylobacterium* species, is of interest because they play an important role in the atmospheric methanol cycle by utilizing methanol as their sole source of carbon and energy (Corpe and Rheem 1989). They also produce plant growth-promoting substances such as cytokinins, auxins, and vitamin B_12_ (Trotsenko et al. 2001, Ivanova et al. 2006) and are involved in seed germination, root development, and increased yield of agricultural plants (Meena et al. 2012).

Members of the genus *Methylobacterium* are pink-pigmented facultative methylotrophs (PPFMs) (Kelly et al. 2014). They utilize one-carbon compounds, including methanol (CH_3_OH), methylamine (CH_3_NH_2_), and formaldehyde (CH_2_O), and multi-carbon compounds containing no carbon–carbon bonds, as well as organic substrates with carbon–carbon bonds as the sole source of carbon and energy (Kelly et al. 2014). They are strict aerobes belonging to the α-*Proteobacteria*, order *Rhizobiales*, and are Gram-negative and rod-shaped organisms. *Methylobacterium* are found worldwide on the leaves of many different plant species, and studies have shown that *Methylobacterium* species composition varies within and between plant species (Balachandar et al. 2008, Knief et al. 2008). However, geographical location has been shown to have a stronger influence than plant species on both *Methylobacterium* and phyllosphere community composition (Knief et al. 2010, Wellner et al. 2011).

The objective of this study was to investigate the diversity of *Methylobacterium* species in the phyllosphere of native New Zealand (NZ) plants, via the isolation of methanol-utilizing bacteria. Amplified ribosomal DNA restriction analysis (ARDRA) was used to select representative isolates for sequencing. This study is significant as the first exploration of the species composition of the genus *Methylobacterium* in the phyllosphere of native NZ plants.

## Materials and methods

### Sample collection and bacterial isolation

Twenty different native NZ plants were selected for isolation of *Methylobacterium* species (Table 1), plants were chosen to represent a diversity of plant types, including trees, shrubs, herbs, ferns, and flax. Leaves (three) from five plants of each plant species were collected in sterile containers, with the majority collected from the campus of the University of Waikato (Hamilton, NZ), where they grow naturally. Leaves collected were healthy but not new growth, and were all collected in the morning. Leaf-imprinting was used to isolate bacteria from the leaf surface (Corpe 1985). Immediately after collection, leaves were laid directly on the surface of 0.5% methanol-supplemented ammonium mineral salt (AMS) agar plates and impressed carefully, large leaves were cut to the desired size and small leaves used whole, plates were then sealed with parafilm, and incubated at 30°C for up to two weeks (Holland and Polacco 1994). AMS agar medium contained: 0.7 g/l K_2_HPO_4_, 0.54 g/l NH_4_Cl, 0.1 mg/l ZnSO_4_.7H_2_O, 0.03 mg/l MnCl_2_.4H_2_O, 0.3 mg/l H_3_BO_3_, 0.2 mg/l CoCl_2_.6H_2_O, 0.01 mg/l CuCl_2_.2H_2_O, 0.02 mg/l NiCl_2_.6H_2_O, 0.06 mg/l Na_2_MoO_4_.2H_2_O, 15.0 g/l Difco^TM^ agar (1.5%), in distilled water, pH was adjusted to 6.8 and the medium sterilized by autoclaving (Whittenbury et al. 1970). An amount of 0.5% methanol was added aseptically to the autoclaved medium upon cooling and mixed thoroughly before the plates were poured. After incubation, colonies were chosen randomly from the plates and streaked. Colonies were re-streaked 5–6 times on fresh AMS agar plates to obtain a pure culture.

**Table 1.** NZ native plant species used in this study.

Scientific name	Common/Maori name	Family
*Agathis australis*	Kauri	*Araucariaceae*
*Alectryon excelsus*	Titoki	*Sapindaceae*
*Asplenium oblongifolium*	Shining Spleenwort/Huruhuruwhenua	*Aspleniaceae*
*Blechnum novea-zealandiae*	Palm-leaf fern/Kiokio	*Blechnaceae*
*Coprosma robusta*	Karamu	*Rubiaceae*
*Cyathea cunninghamii*	Gully tree fern	*Cyatheaceae*
*Cyathea dealbata*	Silver tree fern/Ponga	*Cyatheaceae*
*Cyathea medullaris*	Black tree fern/Mamaku	*Cyatheaceae*
*Griselinia littoralis*	Kapuka	*Griseliniaceae*
*Hebe elliptica*	Kokomuka	*Plantaginaceae*
*Knightia excelsa*	Rewarewa	*Proteaceae*
*Macropiper excelsum*	Kawakawa	*Piperaceae*
*Melicytus ramiflorus*	Mahoe	*Violaceae*
*Metrosideros excelsa*	Pohutokawa	*Myrtaceae*
*Olearia traversii*	Chatham Island Akeake	*Asteraceae*
*Olearia albida*	Tree daisy/Tanguru	*Asteraceae*
*Phormium cookianum*	Mountain Flax/Wharariki	*Hemerocallidaceae*
*Phormium tenax*	Common Flax/Harakeke	*Hemerocallidaceae*
*Pittosporum tenuifolium*	Black Matipo/Kohuhu	*Pittosporaceae*
*Plagianthus regius*	Ribbonwood/Manatu	*Malvaceae*

### DNA extraction from pure cultures

For ARDRA analysis, DNA was extracted from bacterial colonies picked with a sterile toothpick into a 1.5-ml microfuge tube containing 1 ml of sterile H_2_O. The tube was vortexed vigorously until cells were dispersed, boiled for 10 min, centrifuged for 5 min at 1000 rpm, then kept on ice. The supernatant (5 µl) was used as a PCR template.

Prior to the extraction of DNA for sequencing (Marmur 1961), isolates were grown in 50 ml AMS plus methanol broth at 30°C, transferred into 50 ml sterile falcon tubes, and centrifuged for 3 min at 3000 rpm. The supernatant was then removed and the cell pellet resuspended in 400 µl SET buffer (20% sucrose, 50 mM EDTA, 50 mM Tris.HCl). Lysozyme solution (50 mg/ml in TE plus 10 mM NaCl) was added (20 µl) and incubated at 37°C for 1 hour. After incubation, 20 µl of 20% SDS and 10 µl of proteinase K solution (20 mg/ml in TE) were added to the lysates and incubated at 60°C for 3 hours. The digested lysates were then purified by phenol chloroform extraction, concentrated by ethanol precipitation, and DNA was stored at −20°C. DNA concentration was quantified using a NanoDrop^TM^ 1000 spectrophotometer and concentration was adjusted to 50–60 ng/µl with TE buffer.

### PCR amplification and ARDRA of 16S rRNA genes

The 16S rRNA gene was amplified using universal eubacterial primers, 27F (5′-AGAGTTTGATCMTGGCTCAG-3′) and 1492R (5′-TACGGYTACCTTGTTACGACTT-3′). For each isolate, 5 µl of template DNA was added to 45 µl PCR master mix in 200 µl thin-walled PCR tubes. Master mix for each reaction contained 24.75 µl of sterile water, 5 µl of 10x PCR buffer, 5 µl of MgCl_2_ (50 mM), 5 µl of 2 mM dNTPs, 2 µl of each primer (10 µM) (Integrated DNA Technologies, Inc), 1 µl of bovine serum albumin (Promega Corporation, USA), and 0.25 µl of *Taq* DNA polymerase (Invitrogen, NZ). PCR was run on a Bio-Rad DNA Engine® (PTC-200) Thermal Cycler (Bio-Rad Laboratories Inc, Hercules, CA, USA). The thermal cycling conditions consisted of 35 cycles (30 sec at 94°C 30 sec annealing at 60°C, and 1 min 30 sec at 72°C) with an initial denaturation of 2 min at 94°C, and a final extension step of 10 min at 72°C. PCR products were visualized by gel electrophoresis, and product size estimated using a 250-bp DNA ladder (Invitrogen).

ARDRA is an established method for determining taxonomic relatedness between isolates (Fisher and Triplett 1999), which were screened to select representatives for sequencing. The 16S rRNA gene PCR amplicon of each isolate was digested with Rsa I, and the products were analyzed by gel electrophoresis using 2% agarose in 1X TAE buffer. A 1 kb + DNA marker (Invitrogen) was run on every gel to size the restriction fragments. All isolates were compared visually for matching fingerprints and grouped into different restriction types. Representative isolates from each restriction group were selected randomly for sequencing.

### 16S rRNA gene sequencing and analysis

PCR products of isolates selected for sequencing were purified using ExoSAP (Affymetrix, Ohio, USA), according to the manufacturer’s protocol. Sequencing reactions were performed at the Waikato DNA Sequencing Facility with primers 27F and 1492R on an ABI 3130XL (Applied Biosystems). Each 16S rRNA gene sequence was analyzed for closest identities using BLASTN in the NCBI database (Altschul et al. 1990).

## Results

To investigate *Methylobacterium* species diversity on NZ native plants, a total of 20 plant species from different plant types (tree, shrub, herb, fern, and flax) were selected. After 10–14 days incubation of leaf-imprinted agar plates at 30°C, colonies were selected for streaking on fresh plates, and over an extended period, these were re-streaked 4–5 times, resulting in the isolation of 245 pure cultures of methanol-grown strains. The majority of isolates (83%) were pale to vivid pink pigmented, with the remaining isolates either cream (10%) or dark orange to red (7%). Methylotrophs were isolated from the leaves of every plant species, but the number isolated varied between species (Table 2). Most isolates were from *Micropiper excelsum* (21.6%), followed by *P. regius* (20%), *P. tenax* (18.8%), *P. cookianum* (11.4%), and *A. oblongifolium* (7.3%), with the lowest numbers from *B. novea-zealandiae, Cyathea dealbata, C. medullaris*, and *K. excelsa* (0.4%).

**Table 2.** Isolates from each plant species grouped by ARDRA analysis.

	Number of isolates in each OTU																
Plant species	1	2	3	4	5	6	7	8	9	10	11	12	13	14	15	16	Total
*Agathis australis*		3															3
*Alectryon excelsus*	3	2	1						1								7
*Asplenium oblongifolium*	9	3	1		1							1				3	18
*Blechnum novea-zealandiae*		1															1
*Coprosma robusta*		2															2
*Cyathea cunninghamii*	2	1															3
*Cyathea dealbata*			1														1
*Cyathea medullaris*			1														1
*Griselinia littoralis*		4	1														5
*Hebe elliptica*	1	2															3
*Knightia excelsa*		1															1
*Macropiper excelsum*	21	13	8	1										1	2	7	53
*Melicystus ramiflorus*	1	1	2							1							5
*Metrosideros excelsa*		5															5
*Olearia traversii*		3	2														5
*Olearia albida*	3								1	1							5
*Phormium cookianum*		13	7		3				1	1						3	28
*Phormium tenax*	7	27	1				1	2	2	1	1		2			2	46
*Pittosporum tenuifolium*	3	1															4
*Plagianthus regius*	17	25	1	1	1	1				1			2				49
**Total**	67	107	26	2	5	1	1	2	5	5	1	1	4	1	2	15	245

The 16S rRNA gene was amplified from all isolates and analyzed by ARDRA, which resulted in a number of different restriction patterns, reflecting the diversity of methylotrophs isolated. Isolates with identical restriction patterns were grouped into 16 operational taxonomic units (OTUs) (Table 2). The majority of isolates (87.7%) grouped in four OTUs: OTU 2 with 107 isolates (43.7%), OTU 1 (67 isolates, 27.3%), OTU 3 (26 isolates, 10.6%), and OTU 16 (15 isolates, 6.1%). OTUs 5, 9, and 10 each had 5 isolates (2.0%), followed by OTU 13 (4 isolates, 1.6%), OTU 4, 8, and 15 (2 isolates each, 0.8%), and the remaining OTUs each had only one isolate (0.4%).

From the ARDRA analysis, a total of 31 isolates were selected randomly as representatives from each OTU for sequencing. BLAST analysis of the 16S rRNA gene sequences (Table 3) revealed that most isolates (79.6%, 195 of 245) were members of the genus *Methylobacterium*, the majority (54.9%, 107 of 195) belonged to OTU 2 and had 98%–99% identity to *M. mesophilicum* or *M. komagatae*; followed by OTU 1 (34.4%, 67 of 195) with 98%–99% identity to *M. marchantiae* or *M. adhaesivum*; OTU 16 (7.7%, 15) with 97%–99% identity to *M. phyllosphaerae*; OTU 13 (2.1%, 4) with 99% identity to *M. extorquens*; and OTU 4 (1.0%, 2) with 99% identity to *M. marchantiae*. Other α-*Proteobacteria* methylotrophs isolated included 28 (11.4% of total isolates) members of the genus *Hyphomicrobium*, closely related (98%–99%) to *H. facile* (OTU 3 and 15), five isolates (OTU 10) related to *Methylopila capsulata* (86%), and one isolate (OTU 14) related to *Rhizobium endophyticum* (95%). β-*Proteobacteria* isolates included five (OTU 5) closely related to *Methylophilus methylotrophus* (99%), one isolate (OTU 6) closely related to *Ramlibacter ginsenosidimutans* (99%), and one isolate (OTU 7) related to *Alcaligenes faecalis* (97%). Of the remaining isolates, one isolate (OTU 12) was a γ-*Proteobacteria* related to *Xanthomonas translucens* (85%), three *Actinobacteria* isolates (OTU 8) related to *Janibacter melonis* (94%), five *Bacteroidetes* isolates (OTU 9) closely related to *Niastella populi* (99%), and one *Firmicutes* isolate (OTU 11) had 99% identity to *Paenibacillus lautus*.

**Table 3.** BLAST analysis of 16S rRNA gene sequences of isolates from each OTU.

OTU	Isolates (%)	Isolate	Isolation source	BLAST match	ID (%)	Matched bp
***Methylobacterium* isolates**						
1	27.3	kk002	*Macropiper excelsum*	*Methylobacterium marchantiae* AB698705	99	993/1005
		kk040			99	974/988
		rw104	*Plagianthus regius*		99	753/756
		kh130	*Pittosporum tenuifolium*		99	812/824
		cf283	*Phormium tenax*	*Methylobacterium adhaesivum* KF681060	98	656/671
2	43.7	kk034	*Micropiper excelsum*	*Methylobacterium komagatae* AB703238	98	647/666
		kk036		*Methylobacterium mesophilicum* KF573002	99	829/838
		rw080	*Plagianthus regius*		99	316/320
		pk208	*Metrosideros excelsa*		98	809/822
		ss136	*Asplenium oblongifolium*		99	641/649
4	0.8	rw087	*Plagianthus regius*	*Methylobacterium marchantiae* AB698705	99	672/680
13	1.6	rw086	*Plagianthus regius*	*Methylobacterium extorquens* LT962688	99	639/646
16	6.1	kk037	*Micropiper excelsum*	*Methylobacterium phyllosphaerae* CP015367	99	713/723
		kk048			99	606/609
		pk243	*Metrosideros excelsa*		97	586/605
		cf284	*Phormium tenax*		99	934/942
**Other Methylotrophic isolates**						
3	10.6	kk004	*Micropiper excelsum*	*Hyphomicrobium facile* MG846099	99	801/813
		kk063			99	730/739
		ss288	*Asplenium oblongifolium*		99	699/709
5	2.0	rw113	*Plagianthus regius*	*Methylophilus methylotrophus* LC191544	99	703/707
6	0.4	rw083	*Plagianthus regius*	*Ramlibacter ginsenosidimutans* KY649387	99	476/481
7	0.4	cf159	*Phormium tenax*	*Alcaligenes faecalis* DQ379508	97	275/283
8	0.8	cf150	*Phormium tenax*	*Janibacter melonis* JN084150	94	472/502
9	2.0	cia197	*Olearia traversii*	*Niastella populi* AB682649	99	666/675
		ti234	*Alectryon excelsus*			
10	2.0	cia203	*Olearia traversii*	*Methylopila capsulata* AJ634928	86	258/299
11	0.4	cf153	*Phormium tenax*	*Paenibacillus lautus* LT601284	99	676/685
12	0.4	ss132	*Asplenium oblongifolium*	*Xanthomonas translucens* DQ424867	85	584/684
14	0.4	kk035	*Micropiper excelsum*	*Rhizobium endophyticum* NR116477	95	761/803
15	0.8	kk005	*Micropiper excelsum*	*Hyphomicrobium facile* MG846099	98	827/840
		kk022			99	814/824

**Figure 1.**
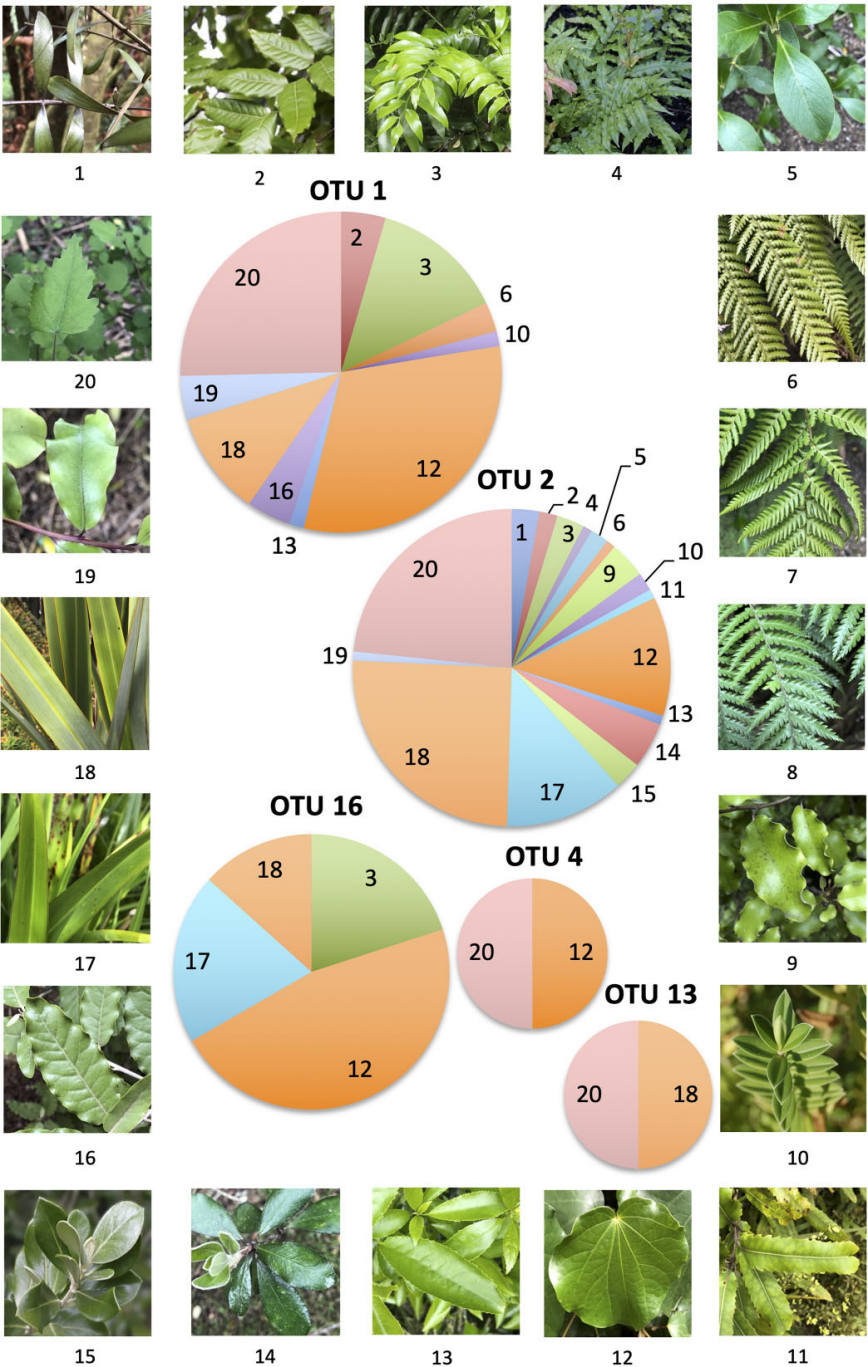
Distribution of *Methylobacterium* isolates from native NZ plants. Pie charts show the number of isolates in each OTU from each plant species, and images show their leaves. The plant species are: 1. *A. australis*, 2. *A. excelsus*, 3. *A. oblongifolium*, 4. *B. novea-zealandiae*, 5*. C. robusta*, 6*. C. cunninghamii*, 7. *C. dealbata*, 8. *C. medullaris*, 9. *G. littoralis*, 10*. Hebe elliptica*, 11. *K. excelsa*, 12. *M. excelsum*, 13. *M. ramiflorus*, 14. *M. excelsa*, 15. *O. traversii*, 16. *Olearia albida*, 17. *P. cookianum*, 18. *P. tenax*, 19. *P. tenuifolium*, 20. *P. regius*.

Most plant species harbored methylobacterial communities of differing complexity (Table 2 and Fig. 1), except the tree ferns *C. dealbata* and *C. medullaris*, from which only *Hyphomicrobium* sp. were isolated. The majority of the *Methylobacterium* isolates, 54.9% (OTU 2), were closely related to *M. mesophilicum* or *M. komogatae*, and they were broadly distributed (17 plant species) and common isolates from most plants. Most isolates were from *P. tenax* (25.2%), *P. regius* (23.4%), *M. excelsum* and *P. cookianum* (12.1% each), *M. excelsa* (4.7%), and *G. littoralis* (3.7%). The second-largest group of *Methylobacterium* isolates (34.4%, OTU 1) was closely related to *M. marchantiae* or *M. adhaesivum* and was isolated from ten plant species. Most isolates were from *M. excelsum* (31.3%), *P. regius* (25.4%), *A. oblongifolium* (13.4%), and *P. tenax* (10.4%). The third group of *Methylobacterium* isolates (7.7%, OTU 16) was closely related to *M. phyllosphaerae* but were only isolated from four plant species, namely *A. oblongifolium, M. excelsum, P. cookianum*, and *P. tenax*, with most isolates (46.7%) from *M. excelsum*. The fourth group (OTU 13) was closely related to *M. extorquens* and was only isolated from two species of plants, *P. tenax* and *P. regius*. The fifth group (OTU 4) was also related to *M. marchantiae* and was only isolated from *M. excelsum* and *P. regius*. No single plant species was found to harbor all of the *Methylobacterium* sp. identified; however, maximum association (with four of the five OTUs) was found with *M. excelsum, P. tenax*, and *P. regius*, followed by *A. oblongifolium* (with three of the five OTUs).

## Discussion

*Methylobacterium* species diversity on the leaves of native NZ plants was investigated using leaf imprinting on methanol-supplemented AMS agar. Methanol is a key substrate for the growth of *Methylobacterium*, and in plants, methanol is produced during plant cell division as the waste product of pectin metabolism (Galbally and Kirstine 2002). A total of 245 methylotrophs were isolated from the leaves of 20 different native NZ plants, but the bacterial species and number of isolates varied between plants. Interspecies variability is frequently seen in other studies of the phyllosphere community (Yang et al. 2001, Lambais et al. 2006, Whipps et al. 2008, Redford et al. 2010). In this study, isolates were selected for sequencing using ARDRA, which identified sixteen different groups of isolates. Sequencing of representative isolates from each restriction group revealed that the majority of isolates (79.5%) were *Methylobacterium* species commonly found to colonize plants (Delmotte et al. 2009, Wellner et al. 2011, Knief et al. 2012a, Knief et al. 2012b). Sixteen different phyllosphere *Methylobacterium* species have been identified (Balachandar et al. 2008, Knief et al. 2008), with *M. extorquens* being a ubiquitous colonizer of the phyllosphere of many plants (Delmotte et al. 2009). This study identified six different *Methylobacterium* species isolated from the leaves of native NZ plants. This low number of species may be because the isolation medium used was highly selective, with only methanol as a carbon source, and *Methylobacterium* species are commonly differentiated according to their carbon utilization ability (Kelly et al. 2014). Another reason could be the degree of association between NZ plants and *Methylobacterium* sp., with other studies showing that *Methylobacterium* association can be epiphytic (Omer et al. 2004), endophytic (Lacava et al. 2004), or symbiotic (Jourand et al. 2004). The *Methylobacterium* species identified in this study (*M. marchantiae, M. adhaesivum, M. komogatae, M. mesophilicum, M. extorquens*, and *M. phyllosphaerae*) are common leaf epiphytes (Knief et al. 2008, Verginer et al. 2010, Schauer et al. 2011, Tani et al. 2012), not surprisingly given that leaf imprinting was used to isolate the bacteria. The final reason could be geographic location, which has been shown to be an important determinant in shaping *Methylobacterium* colonization of the phyllosphere (Knief et al. 2010); however, this could not be addressed in this study.

Another factor to be considered was the effect of leaf texture and structure on the ease of the isolation of bacteria from different plants. The leaves of plants that yielded the most isolates (*P. regius, M. excelsum, P. tenax, P. cookianum*, and *A. oblongifolium*) are thin and easily lie on the medium (see Fig. 1), making imprinting easier than for other plants in this study that have leaves that are thick, glossy, hard, shiny, spiky, or velvety (*A. australis, A. excelsus, C. robusta, G. littoralis, H. elliptica, K. excelsa, M. excelsa, O. albida, O. traversii*, and *P. tenuifolium*). Leaf texture and structure have been shown to be important factors in obtaining epiphytes from the leaf surface using imprinting (Holland et al. 2000). The low number of *Methylobacterium* isolates from *B. novea*-*zealandiae* and *C. cunninghamii* may have been due to the fact that only small, young plants were sampled. But generally, in this study, few *Methylobacterium* species were isolated from ferns, and none were isolated from *C. dealbata* (black fern) or *C. medullaris* (silver fern), which may indicate that either leaf structure, leaf age (fern leaves have a shorter life span than other NZ native plants), or leaf chemistry which may restrict colonization by methylobacteria. However, plant species have been shown to be the main driver for the community structure of *Methylobacterium* species in several studies (Kinkel et al. 2000, Omer et al. 2004, Knief et al. 2008, Knief et al. 2010, Redford et al. 2010, Wellner et al. 2011) and may therefore be significant for NZ native plant species.

The most frequently isolated *Methylobacterium* colonizer was *M. mesophilicum*, which was isolated from 17 plant species and was the only *Methylobacterium* sp. to be isolated from seven plant species (*A. australis, B. novea-zealandiae, C. robusta, G. littoralis, K. excelsa, M. excelsa*, and *O. traversii*), possibly indicating some specialization. Similarly, only *M. marchantiae* was isolated from *O. albida*. From this study, it is difficult to explain the reasons for the association of individual *Methylobacterium* sp. with specific plants. However, plant species and the generalist nature of some *Methylobacterium* species have been found to play a combined role in colonization (Dourado et al. 2012).

Other α-Proteobacteria (*Hyphomicrobium, Methylopila*, and *Rhizobium*), β-Proteobacteria (*Alcaligenes, Methylophilus*, and *Ramlibacter*), γ-Proteobacteria (*Xanthomonas*), Actinomycetes (*Janibacter*), Bacteroidetes (*Niastella*), and Firmicutes (*Paenibacillus*) were also isolated, with similar genera seen in culture-independent studies of the phyllosphere (Jackson et al. 2006, Lambais et al. 2006, Redford et al. 2010, Rastogi et al. 2012). Several studies have demonstrated the association of some of these *Bacteria* with plants, including *Hyphomicrobium* sp. from the *Arabidopsis* phyllosphere (Reisberg et al. 2013), *Methylopila sp*. from banana fruit (Doronina et al. 2013), *R. endophyticum* from the green bean (Lopez-Lopez et al. 2010), and *A. faecalis* from green ash leaves (Sandhu et al. 2009). *Methylophilus* has been found in the phyllosphere (Wellner et al. 2011), *Xanthomonas* sp. have been identified on plant leaves and stems (Corpe and Rheem 1989, Sheng et al. 2011), *Janibacter* have been detected in the rhizosphere (Guiñazú et al. 2013), *N. populi* from the soil of a Euphrates Poplar forest (Zhang et al. 2010), and *P. lautus* from the rhizosphere of wild grass (Sharma et al. 2010). While a number of these genera are known methanol utilizers (*Methylophila, Methylophilus*, and *Hypomicrobium*), to date there is no clear evidence for methanol utilization by most of the other genera of *Bacteria* (*Alcaligenes, Janibacter, Niastella, Ramlibacter, Rhizobium, Paenibacillus*, and *Xanthomonas*), although they did represent only a small number of all the isolates in this study. Further studies are therefore required to understand more about the utilization of carbon substrates by these isolates.

## References

[bib1] Altschul SF , Gish W, Miller W et al. Basic local alignment search tool. J Mol Biol. 1990;215:403–10. 2231712 10.1016/S0022-2836(05)80360-2

[bib2] Balachandar D , Raja P, Sundaram SP. Genetic and metabolic diversity of pink-pigmented facultative methylotrophs in phyllosphere of tropical plants. Braz J Microbiol. 2008;39:68–73. 24031182 10.1590/S1517-838220080001000017 PMC3768351

[bib3] Corpe WA , Rheem S. Ecology of the methylotrophic bacteria on living leaf surfaces. FEMS Microbiol Ecol. 1989;62:243–50.

[bib4] Corpe WA . A method for detecting methylotrophic bacteria on solid surfaces. J Microbiol Methods. 1985;3:215–21.

[bib5] Delmotte N , Knief C, Chaffron S et al. Community proteogenomics reveals insights into the physiology of phyllosphere bacteria. Proc Natl Acad Sci USA. 2009;106:16428–33. 19805315 10.1073/pnas.0905240106 PMC2738620

[bib6] Doronina NV , Kaparullina EN, Bykova TV et al. *Methylopila musalis* sp. nov., an aerobic, facultatively methylotrophic bacterium isolated from banana fruit. Int J Syst Evol Microbiol. 2013;63:1847–52. 22984139 10.1099/ijs.0.042028-0

[bib7] Dourado MN , Andreote FD, Dini-Andreote F et al. Analysis of 16S rRNA and mxaF genes revealing insights into *Methylobacterium* niche-specific plant association. Genet Mol Biol. 2012;35:142–8. 22481887 10.1590/s1415-47572012005000017 PMC3313503

[bib8] Finkel OM , Burch AY, Lindow SE et al. Geographical location determines the population structure in phyllosphere microbial communities of a salt-excreting desert tree. Appl Environ Microbiol. 2011;77:7647–55. 21926212 10.1128/AEM.05565-11 PMC3209174

[bib9] Fisher MA , Triplett EW. Automated approach for ribosomal intergenic spacer analysis of microbial diversity and its application to freshwater bacterial communities. Appl Environ Microbiol. 1999;65:4630–6. 10508099 10.1128/aem.65.10.4630-4636.1999 PMC91617

[bib10] Galbally IE , Kirstine W. The production of methanol by flowering plants and the global cycle of methanol. J Atmos Chem. 2002;43:195–229.

[bib11] Guiñazú LB , Andrés JA, Rovera M et al. Evaluation of rhizobacterial isolates from Argentina, Uruguay and Chile for plant growth-promoting characteristics and antagonistic activity towards *Rhizoctonia* sp. and *Macrophomina* sp. *in vitro*. European J Soil Biol. 2013;54:69–77.

[bib12] Holland MA , Davis R, Moffitt S et al. Using “leaf prints” to investigate a common bacterium. Am Biol Teach. 2000;62:128–31.

[bib13] Holland MA , Long RLG, Polacco JC. *Methylobacterium* spp.: phylloplane bacteria involved in cross-talk with the plant host?In: Lindow SE, Hecht Poinar EI, Elliott VJ (eds.), Phyllosphere Microbiology. St Paul: American Phytopathological Society, 2002, 125–35.

[bib14] Holland MA , Polacco JC. PPFMs and other covert contaminants: is there more to plant physiology than just plant. Ann Rev Plant Physiol Plant Mol Biol. 1994;45:197–209.

[bib15] Ivanova EG , Fedorov DN, Doronina NV et al. Production of vitamin B_12_ in aerobic methylotrophic bacteria. Microbiology. 2006;75:494–6. 17025186

[bib16] Jackson EF , Echlin HL, Jackson CR. Changes in the phyllosphere community of the resurrection fern, *Polypodium polypodioides*, associated with rainfall and wetting. FEMS Microbiol Ecol. 2006;58:236–46. 17064265 10.1111/j.1574-6941.2006.00152.x

[bib17] Jourand P , Giraud E, Bena G et al. *Methylobacterium nodulans* sp. nov., for a group of aerobic, facultatively methylotrophic, legume root-nodule-forming and nitrogen-fixing bacteria. Int J Syst Evol Microbiol. 2004;54:2269–73. 15545469 10.1099/ijs.0.02902-0

[bib18] Kelly DP , McDonald IR, Wood AP. Family *Methylobacteriaceae*. In: Rosenberg E, De Long EF, Lory S, Stackebrandt E, Thompson F (eds.), The Prokaryotes—Alphaproteobacteria and Betaproteobacteria, Vol. 8. Berlin: Springer-Verlag, 2014, 313–40.

[bib19] Kinkel LL , Wilson M, Lindow SE. Plant species and plant incubation conditions influence variability in epiphytic bacterial population size. Microb Ecol. 2000;39:1–11. 10790512 10.1007/s002489900182

[bib20] Knief C , Delmotte N, Chaffron S et al. Metaproteogenomic analysis of microbial communities in the phyllosphere and rhizosphere of rice. ISME J. 2012a;6:1378–90. 22189496 10.1038/ismej.2011.192 PMC3379629

[bib21] Knief C , Dengler V, Bodelier PL et al. Characterization of *Methylobacterium* strains isolated from the phyllosphere and description of *Methylobacterium longum* sp. nov. Antonie Van Leeuwenhoek. 2012b;101:169–83. 21986935 10.1007/s10482-011-9650-6

[bib22] Knief C , Frances L, Cantet F et al. Cultivation-independent characterization of *Methylobacterium* populations in the plant phyllosphere by automated ribosomal intergenic spacer analysis. Appl Environ Microbiol. 2008;74:2218–28. 18263752 10.1128/AEM.02532-07 PMC2292606

[bib23] Knief C , Ramette A, Frances L et al. Site and plant species are important determinants of the *Methylobacterium* community composition in the plant phyllosphere. ISME J. 2010;4:719–28. 20164863 10.1038/ismej.2010.9

[bib24] Korner E , Dahl CC, Bonaventure G et al. Pectin methylesterase NaPME1 contributes to the emission of methanol during insect herbivory and to the elicitation of defence responses in *Nicotiana attenuata*. J Exp Bot. 2009;60:2631–40. 19380422 10.1093/jxb/erp106 PMC2692009

[bib25] Lacava PT , Araujo WL, Marcon J et al. Interaction between endophytic bacteria from citrus plants and the phytopathogenic bacteria *Xylella fastidiosa*, causal agent of citrus-variegated chlorosis. Lett Appl Microbiol. 2004;39:55–59. 15189288 10.1111/j.1472-765X.2004.01543.x

[bib26] Lambais MR , Crowley DE, Cury JC et al. Bacterial diversity in tree canopies of the Atlantic forest. Science. 2006;312:1917. 16809531 10.1126/science.1124696

[bib27] Lindow SE , Brandl MT. Microbiology of the phyllosphere. Appl Environ Microbiol. 2003;69:1875–83. 12676659 10.1128/AEM.69.4.1875-1883.2003 PMC154815

[bib28] Lopez-Lopez A , Rogel MA, Ormeno-Orrillo E et al. *Phaseolus vulgaris* seed-borne endophytic community with novel bacterial species such as *Rhizobium endophyticum* sp. nov. Syst Appl Microbiol. 2010;33:322–7. 20822874 10.1016/j.syapm.2010.07.005

[bib29] Marmur J. A procedure for the isolation of deoxyribonucleic acid from micro-organisms. J Mol Biol. 1961;3:208–18.

[bib30] Meena KK , Kumar M, Kalyuzhnaya MG et al. Epiphytic pink-pigmented methylotrophic bacteria enhance germination and seedling growth of wheat (T*riticum aestivum*) by producing phytohormone. Antonie Van Leeuwenhoek. 2012;101:777–86. 22200783 10.1007/s10482-011-9692-9

[bib31] Omer ZS , Tombolini R, Gerhardson B. Plant colonization by pink-pigmented facultative methylotrophic bacteria (PPFMs). FEMS Microbiol Ecol. 2004;47:319–26. 19712320 10.1016/S0168-6496(04)00003-0

[bib32] Rastogi G , Sbodio A, Tech JJ et al. Leaf microbiota in an agroecosystem: spatiotemporal variation in bacterial community composition on field-grown lettuce. ISME J. 2012;6:1812–22. 22534606 10.1038/ismej.2012.32 PMC3446804

[bib33] Redford AJ , Bowers RM, Knight R et al. The ecology of the phyllosphere: geographic and phylogenetic variability in the distribution of bacteria on tree leaves. Environ Microbiol. 2010;12:2885–93. 20545741 10.1111/j.1462-2920.2010.02258.x PMC3156554

[bib34] Reisberg EE , Hildebrandt U, Riederer M et al. Distinct phyllosphere bacterial communities on *Arabidopsis* wax mutant leaves. PLoS One. 2013;8:e78613. 24223831 10.1371/journal.pone.0078613 PMC3818481

[bib35] Sandhu A , Halverson LJ, Beattie GA. Identification and genetic characterization of phenol-degrading bacteria from leaf microbial communities. Microb Ecol. 2009;57:276–85. 19034559 10.1007/s00248-008-9473-9

[bib36] Schauer S , Kampfer P, Wellner S et al. *Methylobacterium marchantiae* sp. nov., a pink-pigmented, facultatively methylotrophic bacterium isolated from the thallus of a liverwort. Int J Syst Evol Microbiol. 2011;61:870–6. 20495043 10.1099/ijs.0.021915-0

[bib37] Sharma M , Mishra V, Rau N et al. Functionally diverse rhizobacteria of *Saccharum munja* (a native wild grass) colonizing abandoned morrum mine in Aravalli hills (Delhi). Plant Soil. 2010;341:447–59.

[bib38] Sheng HM , Gao HS, Xue LG et al. Analysis of the composition and characteristics of culturable endophytic bacteria within subnival plants of the Tianshan Mountains, northwestern China. Curr Microbiol. 2011;62:923–32. 21061126 10.1007/s00284-010-9800-5

[bib39] Sy A , Giraud E, Jourand P et al. Methylotrophic *Methylobacterium* bacteria nodulate and fix nitrogen in symbiosis with legumes. J Bacteriol. 2001;183:214–20. 11114919 10.1128/JB.183.1.214-220.2001 PMC94868

[bib40] Sy A , Timmers AC, Knief C et al. Methylotrophic metabolism is advantageous for *Methylobacterium extorquens* during colonization of *Medicago truncatula* under competitive conditions. Appl Environ Microbiol. 2005;71:7245–52. 16269765 10.1128/AEM.71.11.7245-7252.2005 PMC1287603

[bib41] Tani A , Sahin N, Kimbara K. *Methylobacterium gnaphalii* sp. nov., isolated from leaves of *Gnaphalium spicatum*. Int J Syst Evol Microbiol. 2012;62:2602–7. 22199216 10.1099/ijs.0.037713-0

[bib42] Trotsenko Y , Ivanova EG, Doronina NV. Aerobic methylotrophic bacteria as phytosymbionts. Mikrobiologiia. 2001;70:725–36. 11785128

[bib43] Verginer M , Siegmund B, Cardinale M et al. Monitoring the plant epiphyte *Methylobacterium extorquens* DSM 21961 by real-time PCR and its influence on the strawberry flavor. FEMS Microbiol Ecol. 2010;74:136–45. 20662926 10.1111/j.1574-6941.2010.00942.x

[bib44] Vorholt JA. Microbial life in the phyllosphere. Nat Rev Microbiol. 2012;10:828–40. 23154261 10.1038/nrmicro2910

[bib45] Wellner S , Lodders N, Kampfer P. Diversity and biogeography of selected phyllosphere bacteria with special emphasis on *Methylobacterium* spp. Syst Appl Microbiol. 2011;34:621–30. 22000032 10.1016/j.syapm.2011.08.005

[bib46] Whipps JM , Hand P, Pink D et al. Phyllosphere microbiology with special reference to diversity and plant genotype. J Appl Microbiol. 2008;105:1744–55. 19120625 10.1111/j.1365-2672.2008.03906.x

[bib47] Whittenbury R , Phillips KC, Wilkinson JF. Enrichment, isolation and some properties of methane-utilizing bacteria. J Gen Microbiol. 1970;61:205–18. 5476891 10.1099/00221287-61-2-205

[bib48] Woodward FI , Lomas MR. Vegetation dynamics—simulating responses to climatic change. Biol Rev Camb Philos Soc. 2004;79:643–70. 15366766 10.1017/s1464793103006419

[bib49] Yang CH , Crowley DE, Borneman J et al. Microbial phyllosphere populations are more complex than previously realized. Proc Natl Acad Sci USA. 2001;98:3889–94. 11274410 10.1073/pnas.051633898 PMC31148

[bib50] Zhang K , Wang Y, Tang Y et al. *Niastella populi* sp. nov., isolated from soil of Euphrates poplar (*Populus euphratica*) forest, and emended description of the genus *Niastella*. Int J Syst Evol Microbiol. 2010;60:542–5. 19654358 10.1099/ijs.0.012112-0

